# Responsible data selection method for algorithmic personalization of health apps: a case study on promoting mental health

**DOI:** 10.3389/fdgth.2026.1691697

**Published:** 2026-06-24

**Authors:** Esra Cemre Su de Groot, Ujwal Gadiraju, Olya Kudina, Loes Keijsers, Manon H. J. Hillegers, Willem-Paul Brinkman

**Affiliations:** 1 Web Information Systems, Delft University of Technology, Delft, Netherlands; 2 Values, Technology & Innovation, Delft University of Technology, Delft, Netherlands; 3 Department of Psychology, Education and Child Studies, Erasmus University Rotterdam, Rotterdam, Netherlands; 4 Department of Child and Adolescent Psychiatry/Psychology Erasmus MC Sophia Children’s Hospital, Erasmus University Medical Center, Rotterdam, Netherlands; 5 Interactive Intelligence, Delft University of Technology, Delft, Netherlands

**Keywords:** algorithms, ethical risks, machine learning, mHealth, personalization, regulations, responsible data selection, trade-off

## Abstract

Digital technologies are on the rise to promote health. To improve the engagement and effectiveness of these technologies, there is a growing interest in algorithmic personalization. However, the user input data for these algorithms (e.g., data from wearables or self-reported data) can come with ethical and regulatory implications. Despite a growing amount of theoretical work, there is no practical precedent on how to consider these implications in the development of personalization algorithms. Therefore, our work aims to tackle this challenge by proposing a stepwise method for Responsible Data Selection (ReDS) for algorithmic personalization of mHealth. The ReDs method acts from a duty of care and promotes an active search for ethically less risky data. We demonstrate the six-step method through a real-world use case on an mHealth app promoting adolescents' mental well-being, using a dataset of 1181 adolescents (5199 interactions) who received coping strategy challenges based on cognitive behavioral therapy. First, we identified the personalization objective in the case study (step 1). The objective was to personalize the type of challenge to promote adherence while diversifying the coping strategy types within the completed challenges. Next, we identified the emotional state of the adolescent and prior completion rates as promising input data (step 2). However, personal emotion data can be considered sensitive, personal, and private, implying ethical implications (step 3). As a potential alternative, tiredness data can be perceived as less sensitive to share and collect (step 4). Subsequently, we analyzed the utility of all data features (step 5) using evaluative simulations with reinforcement learning models. This revealed that solely using the completion rates of the previous day could already benefit the personalization objective and that adding emotion data or tiredness data could similarly further increase the performance of the personalization algorithm. When determining the utility-risk trade-off (step 6), we conclude that tiredness data can be used as an alternative for emotion data if risk mitigation strategies are deployed. Through this case study, we demonstrate the practical utility of the ReDS method. We hope that our work will inspire future developers of personalization algorithms to explicitly incorporate ethical considerations in the algorithm development process.

## 1 Introduction

Digital technologies are increasingly used to promote positive behavior change in health (1, 2). For instance, apps can be used to promote physical activity (3–5), a healthy diet (5, 6), or mental health (7–9). However, sufficient adherence and engagement with these apps are often challenging (10–12), reducing their potential effectiveness. This is often caused by a mismatch between the app and personal needs (12, 13). Personalization has shown a potential to increase engagement and effectiveness by aligning apps towards user needs (14–17). This alignment can already be achieved using relatively simple personalization techniques, such as empowering users to decide what they want, supporting a therapist or developer to decide what is the best option, or devising a rule-based system (18). But besides these techniques, there is a growing interest in using machine learning (ML) algorithms to personalize health apps, such as supervised, unsupervised, and reinforcement learning (18–22).

To personalize health apps to user needs, ML algorithms need input data about the user. Examples of different types of input data that can be used are contextual (e.g., location data, sleep patterns, emotion data, app activity, etc.), physiological (e.g., heart rate, body temperature, breathing rate, etc.), and demographic data (e.g., sex, age, etc.) (21). This data can be collected using passive methods, such as wearable sensors or activity logs, or active self-report methods, such as the experience sampling method (ESM; i.e., questionnaires once or multiple times a day) (23, 24). Furthermore, input data can also be inferred from passive measurements using ML predictive models. For instance, sensor data from wearables can be used to detect emotions (25). Often, a combination of several data types is used as input for the personalization algorithms.

Understandably, continuous or repeated user monitoring for sufficient input data for the personalization of health apps comes with ethical and regulatory challenges, depending on the use case and context. Monitoring of data that relates to a user’s mental or physical state can be experienced as sensitive and personal by the user, which may impose privacy risks (26–28). In this regard, Valentine et al. (26) describe the *privacy/personalization trade-off*; more user information is necessary to foster personalization, in exchange for some of the user’s privacy. It is then important to assess the associated privacy risks against the potential benefits of personalization. In addition, while self-monitoring can promote a sense of autonomy and self-insight, it may also increase the burden on the user. For instance, repeatedly confronting the user with a mental health condition can be perceived as burdensome (27, 29) and lengthy ESM questionnaires as cumbersome (30). Inferring data using passive sensing methods, such as wearables, may reduce the burden on the user in this regard. However, other ethical or regulatory risks may arise. For instance, detecting emotions based on data from wearables can be incorrect due to flawed or imperfect technology, or biases in the emotion recognition system (31). Therefore, using the derived emotion from a biased emotion recognition system would be unfair to those for whom the system does not work well. Furthermore, the EU AI act (32)—adopted by the European Council in 2024 to regulate artificial intelligence (AI) systems—categorizes “emotion recognition systems” as high-risk systems. This suggests that health apps using such a system should undergo a risk assessment before they can be deployed.

While there exists a body of work specifically focused on the ethical considerations of algorithmic and data-driven technology for health apps (26, 27, 33–35), Gooding and Kariotis (36) identified a gap between those studies and more applied empirical research that actually uses algorithmic and data-driven technology in mental health. Similarly, for general health recommender systems that promote healthy behaviors, De Croon et al. (37) reported that most studies score very low on reporting ethical issues. Thus, applied technical studies do not often report *why* and *how* ethical decisions in the development process are taken.

Explicit reporting on ethical considerations when selecting data for personalization algorithms is important for two reasons. First, it forces developers/researchers to think critically about the ethical implications of collecting and using specific data upfront and during algorithmic development, which is essential if the tool will be deployed in a real-life setting (38). Second, explicit reporting on ethical considerations and decisions can increase awareness of the context of development and use, and inform and inspire other researchers and developers to make ethical decisions in similar use cases. Some examples of existing methods that can stimulate the reporting of reflections on developed models and data are model cards (39), the data nutrition project (40), datasheets for datasets (41), and the cognitive bias checklist (42). Specifically for recommender systems, a user-centric ethical recommendation framework has been proposed (43), as well as a framework to detect ethical issues in each data processing stage and suggest implementations accordingly (44). These methods can promote ethical reflections and transparency on the data and models used, and may provide guidance on how to deal with more sensitive data and mitigate their ethical risks.

However, they do not necessarily practically guide ethical decision-making for data selection. Essentially, the aforementioned existing methods (39–44) mainly focus on ethical reflection and how to deal with ethical considerations related to the data given that the data is collected, used, and useful for the personalization objective. They do not guide whether to even collect the data in the first place, include the data in the algorithm, nor stimulate the exploration of alternative data features. To our knowledge, there are no clear practical precedents on how to incorporate a broad perspective of ethical considerations directly into the data selection decision-making process as part of the development of personalization algorithms for promoting health.

As a first step towards bridging the gap from ambition to action, we propose a practical and responsible stepwise method for selecting data for personalization algorithms—*Responsible Data Selection (ReDS)*. This method allows for explicit consideration of ethical considerations in deciding which data to select as part of the development of personalization algorithms for health apps. With health apps we refer to software programs on mobile devices that process health-related data for their users, according to the definition given by Maaß et al. (45). These apps aim to promote health or well-being, diagnose and treat acute health issues, rehabilitate and manage chronic disease, or provide long-term care. Our ReDS method is guided by the duty of care (46), which states that developers of AI systems are responsible for avoiding any harm caused by the system. In doing so, we focus on mitigating data-associated risks when selecting input data for algorithmic personalization. This does not only mean considering *what* data has *which* ethical implications, but more importantly, understanding *why* the specific data is necessary for the personalization objective, and finding ethically less risky alternatives. Next, the ethical consequences of choosing certain data need to be considered before implementation into a real-world health app (i.e., what their collection and use would imply).

To illustrate and operationalize the *ReDS* method in a real-world setting, we used a case study, which is a common method to evaluate frameworks (47). The aim of the use case is to select input data for the algorithmic personalization of daily challenges for adolescents, which are based on cognitive behavioral therapy (CBT) and aim to promote several coping strategies. The personalization algorithm should decide which coping challenge should be offered in a specific moment, based on the state of the adolescent. Here, we identify the ethical risks of using emotion data for the personalization objective and explore alternative options, such as using tiredness data and/or prior completion rates. Using this case study, we demonstrate how the presented method can be used and lead to useful results. We hope to inspire future designers and developers of personalization algorithms for health to seek responsible data options proactively. The main contributions of our work are twofold:

1. We propose a novel stepwise method for responsible data selection called *ReDS* for algorithmic personalization of health apps. The method promotes explicit consideration and reporting on ethical and regulatory risks in the algorithm development process.
2. We demonstrate a practical precedent for following the ReDS method by applying it to a real-world use case.

## 2 Related work

### 2.1 Mitigating data-related risks in health apps

Researchers have discussed several ethical issues that may relate to the data used and stored by health apps and suggested ways to mitigate the associated risks (26–28, 33). One of these issues is harmful biases, such as biases related to race, sex, and age (34, 38, 48). These biases in the data can cause the algorithm to act unfairly. For instance, an underrepresentation of individuals with certain characteristics might cause the algorithm to perform inadequately for those individuals. In this regard, developers of algorithms should use diverse and representative datasets, be aware of potential biases, and take mitigation strategies to promote fairness (34, 49).

Another data-related ethical issue in health apps relates to user privacy, and suggested solutions often consist of implementing transparent informed consents (27, 28, 33). This includes explicit communication to the user about what data is collected, why it is being collected, and how it is stored (27). Additionally, researchers made suggestions to include granular consents (27, 50). With granular consent, users can decide for themselves which data they would like to share (51). Opting out of sharing specific information might impact the quality of the personalization, according to the privacy/personalization trade-off (26). In that case, it might be transparent to communicate to the user the effect of not sharing specific parts of the data on the personalization quality. These suggested methods related to data privacy have a commonality in that they all aim to promote user autonomy in making an informed decision on whether they would like to engage with the system and share their data.

There exist several regulations that are relevant to mitigating data-related risks in the development and deployment of health apps. In Europe, the GDPR (52) protects the data and rights of users by law. For instance, by being transparent about what data is being used and why, and providing users with the rights to see, correct, or delete their data. Furthermore, GDPR only allows processing of special categories of data (e.g., race, political opinions, or health data) under specific circumstances, for example, when using explicit consent, or to protect vital interests of the data subject. When apps can be considered a medical device, they should also comply with the medical device regulations (MDR) (53) as set by the European Parliament and Council. Notably, not all health apps may be considered medical devices (e.g., when promoting well-being). Furthermore, when personalization algorithms are used, one should comply with the EU AI act (32), which aims to ensure all AI systems are safe, transparent, traceable, non-discriminatory, and environmentally friendly. In the United States, the US Health Insurance Portability and Accountability Act (HIPAA) (54) aims to protect medical and health data and the privacy of patients. For the data selection process specifically, these regulations thus mandate transparency and justification on what data is being collected and for what purpose, with specific care for special types of data. However, they do not provide a step-wise approach to incorporate ethical data-related considerations in the data selection process. With our proposed method for responsible data selection for personalization algorithms of health apps, we aim to stimulate ethical reflections that include but also move beyond these regulations and stimulate the exploration of more responsible data-related choices—something that is not explicitly stimulated by regulations.

### 2.2 The privacy paradox of the users

While many of the above-mentioned methods increase user autonomy relating to privacy-related decisions, sometimes users’ decisions on sharing personal data do not align with their privacy concerns. This is called the privacy paradox. Gerber et al. (55) outlined several explanations for the privacy paradox. Social influence may play a role; if others are sharing data, you might do this as well (55, 56). Furthermore, trust in the data processor may cause people to share information, even if they have privacy concerns relating to the personal data itself (55, 56). Choi et al. (57) also explained how privacy behavior can be affected by privacy fatigue. Privacy fatigue relates to feeling weary about making decisions on online privacy, often due to the difficulty of making these privacy-related decisions. This may also relate to the difficulty of understanding the informed consents (58). This notion can cause users to disclose personal information, despite having privacy concerns. Lastly, the most well-discussed explanation is the privacy calculus model (59), which states that if the expected benefits of data sharing outweigh the anticipated privacy-related risks, one is willing to share personal data.

However, there are ways to empower the user in making these privacy-related decisions according to their values and potentially overcome the privacy paradox. For instance, Lee and Kwon (60) developed a feature selection method to solve the personalization-privacy paradox in mobile wellness healthcare services, based on the privacy calculus model. Their proposed feature selection model finds a subset of features while taking into account users’ privacy concerns relating to the features and the performance of the personalization. This way, the model automatically deploys the privacy calculus model. Another way to empower users is to use nudging to assist users in making privacy-related decisions (61). For instance, by clarifying what the risks and benefits are when sharing information as part of the design of the interface in the app. The review of Acquisti et al. (61) shows an overview of several ways in which nudges can be used to enhance user choices relating to privacy and security.

### 2.3 The developers’ responsibility

While all aforementioned methods empower the user in making data privacy-related decisions, these methods work on the assumption that the user is able to accurately estimate privacy-associated risks (with or without the help of nudges). In reality, people’s estimates of the associated risks can be influenced by, for example, cognitive biases (55, 56). An example of such a cognitive bias is an *affect bias*, which can cause people to underestimate the risks of something they like and overestimate the risks of something they dislike (62). Furthermore, users may lack the metacognitive ability (63), knowledge, and experience to make an informed estimate of privacy risks (55, 56). In line, a recent study by Korneeva et al. (64) found that users’ privacy literacy relates to the ability to detect privacy issues. While nudging techniques may help in mitigating some of these cognitive biases, the appropriate technique might differ per user and context, making it a challenging task (61). In short, we cannot simply assume that all users are willing and capable of making estimates on the data-associated risks. Therefore—without disregarding the autonomy of the users—our proposed method focuses on the responsibility of the developers’ side to proactively make ethical considerations when selecting data for algorithmic personalization.

### 2.4 Assessing ethical considerations

Efforts have been made to quantify specific data-related ethical concerns on the developer’s side with the goal to optimize and automate the data selection process for machine learning algorithms (65–67). For instance, Belitz et al. (65) have used six statistical unfairness definitions to balance the fairness of specific data features with model accuracy. This way, their feature selection method dealt with the trade-off between fairness and accuracy. Furthermore, Kil et al. (66) proposed an optimal feature selection mechanism that optimizes the privacy-utility trade-off in memory-limited environments. Im et al. (67) explored the tradeoff between data privacy in terms of data de-identification and utility, using a clinical data use case. They found that de-identification compromised data utility and concluded that it is difficult to maintain both high privacy and utility. Furthermore, Sambasivan et al. (68) highlighted the compounding events that cause negative, downstream effects from data issues. They showed that such effects are triggered by conventional practices in machine learning that undervalue data quality. Others have explored the use of fairness toolkits that aim to support practitioners in using algorithmic fairness metrics and harm mitigation methods, revealing a plethora of human and organizational factors that shape how such tools are designed, deployed, and used (69, 70). Recent work in trustworthy AI and governance has argued for broadening the myopic lens through which ethical considerations are typically made, and to explicitly consider the entire breadth of AI supply chains (71, 72). Moving from these valuable conceptual and theoretical guidelines to actionable practices remains an unresolved challenge.

Complementing existing efforts, we aim to incorporate a broader ethical perspective into the data selection process, for which an automated feature selection may not be the right method for two reasons. First, a recent review by Palumbo et al. (73) identified a lack of quantitative measures to capture a variety of ethical principles for ethical AI. They identified a strong focus on fairness, diversity, and non-discrimination metrics, while there is a lack of metrics for other ethical principles (i.e., human agency and oversight, technical robustness and safety, privacy and data governance, transparency, social and environmental well-being, and accountability), making it difficult to implement the full range of ethical principles in the data science pipeline (e.g., in the data selection process). Second, other work discusses whether ethics should even be quantitatively measured in the first place (74). For instance, LaCroix and Luccioni (75) argue that it is impossible to have a way of measuring the ethics of an AI system and assessing its moral correctness. Such a measure and assessment would imply that there are underlying static values that should be met, while in reality, values are situated, responsive to the sociomaterial framing, and hence may change (76). Furthermore, the abstraction of ethical principles or values into metrics risks the removal of the (social) context that shapes the ethical understanding and reflection (77). We want our method to be flexible in considering various ethical principles, values, and regulations, while also allowing for flexibility to consider and account for the entire sociotechnical context. Therefore, we deliberately chose not to use metrics to capture data-related ethical principles. Instead, we aim to systematically stimulate the use of human judgment to allow for deeper ethical reflections, not restricted by the boundaries of a metric.

## 3 Proposed method

**Figure 1:**
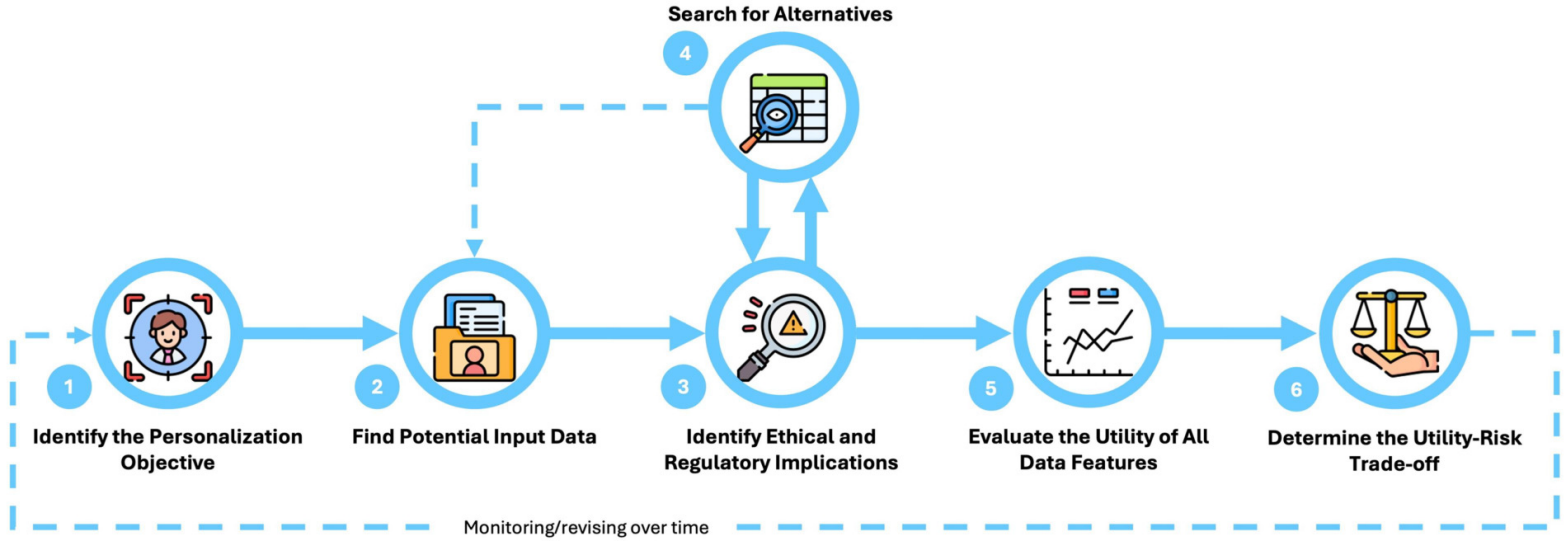
A flowchart of the ReDS method. The dotted arrow between steps 4 and 2 is indicative of alternative data that could be considered for candidate features from step 2. The dotted line between steps 6 and 1 describes the iterative nature of the method. Icons from Flaticon: “View”, “Target Audience”, “User free”, “Identify”, “Line Chart” and “Law” by Freepik, licensed under Flaticon License.

Our method for mitigating data-associated risks and explicitly fostering ethical considerations in the data selection process acts from the duty of care (46), considers the privacy personalization trade-off (26, 78) and the EU AI Act (32). The EU AI Act (32) and its corollary AI Liability Directive (46) establish legal responsibility for AI companies to prevent any harm resulting from the use of AI systems and to ensure consistency with fundamental human rights. As stated and described by Article 9 of the EU AI Act (32), high-risk AI systems require an iterative risk management system, where risks are assessed, and appropriate risk management measures need to be designed and adopted to address the identified risks. Although personalization algorithms for health apps are not necessarily high-risk AI systems, we draw inspiration from this assessment to operationalize the duty of care (i.e., proactively reducing risks of harm). To this end, we consider the privacy-personalization trade-off (26, 78) in the data selection process for the algorithm. In doing so, we believe that if there are data-related ethical issues, it is morally right to search for better alternatives, deploy meaningful mitigation strategies, or assess the risks of not deploying any personalization against the data-related ethical risks. Therefore, we suggest the following steps (visualized in Figure 1) as part of our iterative Responsible Data Selection (ReDS) method for algorithmic personalization:

1. **Identify the Personalization Objective.** The first step is to obtain a clear understanding of the algorithmic personalization objective. *What* needs to be personalized and *why*?
2. **Find potential input data.** Second, potential data features that might be relevant and useful for the personalization objective need to be found. Often, specific features might be considered relevant based on prior research.
3. **Identify ethical and regulatory implications.** Next, it is important to reflect on the ethical and regulatory implications of the potential input data to get an understanding of the data-associated risks that are involved. These reflections may go beyond looking at what is possible from a regulatory perspective or the suggestions made by a university ethics board (33). The ethical and regulatory implications may relate to the collection of the feature (e.g., privacy risks) and/or the use of the feature in an algorithm (e.g., data biases). Furthermore, different types of data—as well as the way in which the data is being collected—may correspond to different risks. For instance, users may not fully be aware of the data being collected and used when using passive sensing data (79, 80) or biases may occur when features are derived from other measures (31, 79). To identify the ethical and regulatory implications of specific data, existing literature can be leveraged, as well as involving domain experts and/or end-users in these reflections.
4. **Search for alternatives.** If the third step resulted in ethical or regulatory risks relating to the prospective input data, we search for alternative data features with less associated ethical and regulatory risks—which thus also requires the identification of ethical and regulatory risks similar to step 3. This data might be less straightforward to use based on prior research, but still potentially useful—based on research, grounded in intuition, or user evaluations on their perception of the associated risks. These alternatives may already be part of the initially available or foreseen data from which the potential input data features from step 2 were selected, but might have been less straightforward to use. Or it might be necessary to collect additional data or augment available data with other existing data. It is possible that—even after an extensive search—no suitable alternative data features are found. In this case, the utility of the features from step 2 can be evaluated in step 5.
5. **Evaluate the utility of all data features.** In this step, we evaluate the utility of the potential data features that are identified in steps 2 and 4 for the personalization objective. If no alternative features were found in step 4, the evaluation focuses on the data features from step 2. The evaluation also needs to be compared against a baseline situation (e.g., having no personalization, or personalization without the use of algorithms). The utility (wherein the appropriate measure depends on the objective) can be evaluated using simulations, empirical experiments in a research setting, or computational experimentation. While simulations may not fully capture real user behavior as opposed to real-world empirical testing, simulations do allow for faster, cost-effective, and safe utility testing. Examples of model-agnostic ways to asses the utility of a data feature are permutation feature importance, leave one feature out (LOFO) importance (81), or using an exhaustive feature selection search (i.e., trying all possible feature combinations). Furthermore, if prior research has already clearly shown the utility of the data feature, this might be used for the evaluation and no further testing is necessary.
6. **Determine the utility-risk trade-off.** Based on the utility of the data for the personalization objective and the associated ethical and regulatory risks, we can make a trade-off to decide what data features will be used for the personalization. For instance, if using the alternative data features results in similar personalization quality, it makes sense to use the alternative features as they have fewer associated risks. However, if the original input data outperforms the alternative features, or if no alternative features are found, a trade-off needs to be made between the data-associated risks and the risks of not deploying any personalization. This trade-off may also consider potential meaningful risk mitigation strategies, such as additional security measures, bias mitigation strategies, or giving autonomy to the user to decide upon the trade-off (e.g., with granular consent). Additionally, conclusions to not operationalize any algorithmic personalization can also be drawn based on the utility-risk trade-off.

## 4 Case study: personalizing coping strategies among adolescents

We demonstrate the ReDS method by operationalizing it in a use case on personalizing coping strategy challenges for adolescents. To this end, we used an existing gamified mHealth app for adolescents, the Grow It! app (82, 83). It was developed to self-monitor daily thoughts, behaviors, and emotions using the Experience Sampling Method (ESM), as a part of which adolescents received 5 questionnaires a day. The app aims to promote adaptive coping by offering daily CBT-based challenges related to one of the coping strategies: acceptance, distraction, problem-solving, or social support. Each day, adolescents could choose between 3 different challenges related to the same coping strategy.

### 4.1 Identify the personalization objective

The aim is to personalize which type of coping strategy challenge should be offered when and to which adolescent. By doing this, the aim is to improve adherence to the challenges, which relates to the effectiveness of mHealth apps (84, 85), particularly over the long term (86). Furthermore, the flexibility in deploying different types of coping strategies depending on the situation contributes to mental resilience (87–89). Therefore, adolescents should expand their skill sets with different types of coping strategies. So, besides promoting adherence, a diversity of coping strategy types within the completed challenges should be promoted. Personalization by facilitating adolescents to freely choose the coping strategy challenge they wish to complete would not fit this personalization objective. This could breed grounds for adolescents to stick with their favorite type of coping strategy challenge and neglect others, which does not contribute to expanding their coping skill set. Therefore, the aim is to explore whether algorithmic personalization may help with the personalization objective.

Reinforcement learning (RL) algorithms can adaptively personalize to individual preferences and behaviors (90) and have also shown potential for improving engagement or health-related outcomes by personalizing health behavior change interventions (22). RL algorithms are able to personalize a sequence of actions based on the state of an individual. And when deploying them in practice, they can adapt to changes over time. Therefore, we used an RL algorithm to personalize the type of coping strategy challenge based on the adolescents’ states, while promoting adherence and diversity of coping strategies.

### 4.2 Find potential input data

To personalize the type of coping challenge based on adolescents’ states, we need to find useful data to describe the dynamic state of the adolescent. The ESM component of the Grow It! app measured several emotions throughout the day, using 7-point Likert-scale questions from the Positive and Negative Affect Schedule (PANAS) (91, 92). The measured positive emotions consisted of *relaxed*, *satisfied*, *happy*, and *confident*. The measured negative emotions consisted of *angry*, *nervous*, *annoyed*, and *sad*. Emotions are known as a strong driver of human behavior (93, 94), and fluctuations in mood can impact decision-making (95). For similar reasons, emotions have been used as input data for recommender systems (96, 97). Also, when deciding which behavior change activity to offer, considering a person’s emotional state is beneficial (98). Furthermore, mood and emotions have been used to describe the states within RL algorithms to personalize health interventions (99, 100). Moreover, Paredes et al. (99) used positive and negative affect specifically from the PANAS to personalize micro-interventions to reduce stress. Altogether, using positive and negative affect measures from the ESM data seems like a straightforward choice when describing adolescents’ states to personalize the coping strategy challenges.

Furthermore, Weimann and Gißke (22) showed that system-use adherence variables are often used to describe the state space in RL algorithms for personalizing health behavior change interventions. The rationale behind this is that individuals who are already active in engaging with the challenges may be more motivated to do the challenge, which might relate to different optimal actions. Therefore, besides using the emotional state, another state feature could describe whether the adolescent is active in completing challenges.

### 4.3 Identify ethical and regulatory implications

The processing of emotion data comes with ethical and regulatory challenges (94, 101–104). Emotions are considered to be personal as they provide insight into one’s mental state and relate to personal values and identity (105). Therefore, people might not feel comfortable or have privacy concerns with sharing their emotions (106). Additionally, asking adolescents about their emotions can be experienced as confronting by some adolescents (27, 107). This might also be the case when presenting explanations to the adolescents that explain why they are receiving a certain challenge suggestion, which is favorable if the system needs to act transparently and promote user autonomy (26, 34). And lastly, if an adolescent reports repeatedly on negative emotions, it might indicate a mental disorder. It would then be morally right to act upon this information.

From a regulatory perspective, the current European General Data Protection Regulation (GDPR) does not necessarily consider emotion data as special data (52, 101), although some scholars have argued that it should be treated like that due to its sensitive nature (101, 103). The EU AI act (32)—adopted by the European Council in 2024 to regulate artificial intelligence (AI) systems—categorizes “emotion recognition systems” as high-risk systems. Notably, there is an exception for the classification of emotion recognition systems as high-risk systems for medical cases (32). However, the context of our use case is not a medical case, as it relates to the general population. Furthermore, the use case might not necessarily be considered an emotion recognition system, as it does not use biometric data to infer emotion, which is how the EU AI Act defines an emotion recognition system (32). Yet, the way the EU AI Act regulates emotion recognition systems does highlight the sensitive nature of emotion data. Altogether, an alternative—less sensitive—option to use as input data for the personalization of challenges to promote adolescents’ coping strategies would be welcome.

Potential ethical risks related to storing the activity of adolescents may relate to the user autonomy and ownership of usage history data. It can be argued that users should have the right to change or delete their usage history (26). Valentine et al. (26) argue that this is particularly the case for medical health apps, as the usage history can then be considered a medical record. As our use case does not relate to a medical situation, we consider this risk to be rather small. Furthermore, this risk may apply to all user data that is collected. For this feature in particular, we consider implementing a risk mitigation strategy that allows users to delete their data as sufficient to handle this risk.

### 4.4 Search for alternatives

A potential alternative measure that has been measured by the ESM questionnaires is fatigue. Fatigue measurements, such as tiredness, might feel less sensitive, private, and close to one’s values and identity compared to emotions. In line, the study of van der Mee et al. (108) found that university students disclosed their tiredness levels almost twice as frequently compared to 25 other emotions or mental states. Furthermore, fatigue measurements have also been explicitly mentioned in the AI act as not falling under the high-risk category (32)—implicating less associated risks compared to emotions. Circling back to the personalization of preventive interventions, tiredness may also influence the likelihood of completing a specific type of intervention, similar to emotions. Corresponding to this thought, a prior study found that sleep quality—which relates to fatigue and tiredness—is associated with the deployment of different stages of behavior change (109). Furthermore, the user’s level of energy has been used to describe the state space for RL algorithms for smoking cessation interventions (110).

Notably, using fatigue measurements does not exclude any ethical risks at all and can still be considered sensitive. For instance, if an adolescent is extremely tired each day, it might be an indication of a mental (e.g., a symptom of depression) or physical disease. However, we consider this risk to be relatively small, as our use case targets the general population of adolescents in a non-medical setting. However, a risk mitigation strategy might be necessary when using tiredness as input data for the personalization objective. Thus, accompanied by a risk mitigation strategy, fatigue measures may be a useful alternative for emotion data. Within the Grow It! app, tiredness was measured by asking adolescents to rate how much they relate to the statement “*I am tired*”, using a 7-point Likert scale.

### 4.5 Evaluate the utility of all data features

To make an informed decision on what data to use to describe the state of the adolescent, we evaluated the utility of the potential data features against a baseline situation with no personalization (i.e., randomly assigning the coping strategy challenges). This was done by creating two different reinforcement learning algorithms, one using emotion data and one using tiredness data (which will both be described as an *ESM feature*). Additionally, the separate elements of the model were evaluated with different RL models: (1) using only the activity feature (i.e., if the adolescent completed a challenge the previous day), (2) using only the ESM feature, and (3) using the activity feature and the ESM feature (we call this the *full model*). Furthermore, the full model was tested without taking future states into account (a myopic RL model) and while considering future states (a non-myopic RL model). This was done to measure the added value of taking the sequence of challenges into account, instead of solely relying on the context of a given moment (which is essentially a contextual multi-armed bandit). Lastly, all these models were compared to the situation without personalization: randomly allocating challenges to adolescents, as this was the initial situation within the mHealth app of our case study. We evaluated these models using simulations based on the existing data, an established method in health research (111). The simulation consisted of 28 timepoints (representing 28 days) on which one challenge was suggested, which could either be completed or not. The utility was measured based on the total adherence (i.e., completion rate) and the diversity of coping strategies within the completed challenges, reflecting the personalization objective.

#### 4.5.1 Dataset description

In this work, we used the dataset of the Grow It! study conducted by Dietvorst et al. (82), approved by the Medical Ethics Committee of the Erasmus Medical Centre (MEC-2020-0287). During the study, a total of 2,974 adolescents (12-25 years) engaged with the Grow It! app within three different cohorts during the COVID-19 pandemic. The first cohort used the Grow It! app for six weeks, and the other two cohorts for three weeks. Details about the implementation of the Grow It! app can be found in the study of Dietvorst et al. (82), and their additional codebook^1^ describes more details on the measurements. We used ESM measures related to emotion and tiredness. In the ESM dataset, the adherence to the ESM was 17.68% and 29.51% to the daily challenges.

#### 4.5.2 Reinforcement learning algorithm

Our method can be described by a Markov Decision Process (MDP) with a tuple of (S,A,T,r,γ). Here, S describes the different states, A describes the actions, T describes the probability of moving from one state to another given a specific action, r describes the reward given a state-action pair, and γ describes the discount factor to favor immediate rewards over long-term rewards. The goal is to derive an optimal policy π that optimizes the action that should be taken in a given state to maximize the total discounted expected reward. The discount factor γ was set to 0.7 to favor rewards in the near future. We used the Gauss-Seidel value iteration algorithm from the Python MDP Toolbox (112) to solve our MDP problem.^2^

##### 4.5.2.1 Data preparation

To solve our MDP problem, the data needed to be prepared into interaction samples of (s0,a,r,s1) tuples. One interaction sample describes the state of the adolescent before the action (s0), the type of coping strategy of the suggested challenges (a), completion of the challenge (while also promoting a diversity of coping strategies in the completed challenges) (r), and the state of the adolescent after the challenge (s1).

To decide s0 and s1, we used the time stamps of the adolescents submitting the results of the challenges to the Grow It! app. For s0, the most recent ESM measure before submitting the challenge was used. This measure had to be on the same day and at least 20 min before submitting the challenge, as most challenges required adolescents to actually *do* something. The ESM data used for s1 consisted of the first ESM measurement after completing the challenge, up to one day after completion. If the challenge was not completed, s1 was described by the evening ESM measurement of the current day, or a measurement on the next day; s0 was described by the first measurement of the current day. Additionally, for 253 adolescents, the submission of the challenges was not stored correctly due to technical issues, making their data unreliable. Therefore, these adolescents were removed from the data. Furthermore, adolescents in Cohort 1 did not receive preventive interventions for the first 7 days of playing the Grow It! app, similar to the last three days of Cohort 2 and 3. Therefore, these days were not included in the data. This resulted in a dataset of 5,199 interaction samples across 1,181 adolescents.

##### 4.5.2.2 Action space

The action space consisted of the different categories of coping strategy challenges: distraction (DI), acceptance (AC), problem-solving (PS), and social support (SS) (82). Therefore, the action space is described as a∈{DI,AC,PS,SS}.

##### 4.5.2.3 State space

The state space consisted of three different elements. The first element is deterministic by nature and is used to track how many of the four different coping strategy challenges (i.e., the actions) have been completed by the adolescent so far. The completion count of these actions was represented by four numerical features. The completion counts (C) tracked the number of completed challenges of a specific coping strategy (action type) up to 4 over time. Therefore, these features can be described as $C_{a}\in \{0,1,2,3,4\}$, where a count of 4 can represent the completion of 4 or more actions, and a describes the action type. The second element is a binary state feature that describes whether the adolescent completed the action the day before. We refer to this feature as the *Preday* feature, which can be described as Preday∈{no,yes}. The third element relates to the ESM measurement, which describes either the emotional state *or* the tiredness state of the adolescent. For the emotional state, the affect balance (AB) was used, which represents the dominance of positive affect over negative affect by subtracting negative from positive affect (113), and accounts for extremity bias (114). For the tiredness state, a single 7-point Likert scale question asking adolescents to indicate how much they relate to the statement “I am tired” was used. To reduce the size of the state space, the AB and tiredness measurements were both divided into four equally sized bins. Therefore, the ESM state feature was described as $ESM_{type}\in \{0,1,2,3\}$, where type either described the tiredness state or the emotional state, and 0 related to not feeling tired or experiencing greater positive affect over negative affect. Altogether, there were 5,000 possible states for the states with affect balance and 5,000 possible states for states including tiredness. These states can be described as $\left(C_{DI},C_{AC},C_{PS},C_{SS},Preday,ESM_{type}\right)$.

##### 4.5.2.4 Reward

The reward needed to promote two objectives: (1) the number of challenges completed (*adherence*) and (2) a variety of different coping style strategies within the completed challenges over the course of the simulation (*diversity*). The first part of the reward function ($r_{1}$) promoted adherence and was set to 0 if the challenge was not completed and 1 if it was completed. The second part of the reward function ($r_{2}$) promoted diversity by adding a cost for action types that were already completed more often, described as $\left(1-1/5*C_{a}\right)$, where 5 represents the number of possible values for $C_{a}$. This cost function uses the state features that track how many of each action type have been completed ($C_{a}$). As a result, action types that have not been completed frequently are favored over actions that are completed multiple times. Therefore, the total reward function for a state-action pair can be described as $r=r_{1}*r_{2}$.

##### 4.5.2.5 Transition function

The transition probabilities for the ESM features ($p_{a}$, where $a=ESM_{type}$) were directly derived from the data, as we assumed that transitions were independent of other state features. The transition probabilities ($p_{b}$) for the action track state features ($C_{a}$) and the Preday state feature depended on the probability of completing the suggested action given the current state and the action. To measure the probability of completing a challenge, the data from the first day of the study was not taken into account. In the original dataset, adolescents all received the same sequence of coping challenges. Therefore, including data from the first day would skew the completion rate for the *Preday* feature towards the suggested action on that first day (as on the first day, none of the adolescents have completed a challenge the day before). The probability of transitioning to a state where the action has been completed can be described as $p_{a}*p_{b}$, and the transition to a state where the action has not been completed as $p_{a}*(1-p_{b})$.

##### 4.5.2.6 Simulation evaluation

The optimal policies resulting from the RL algorithms were evaluated using human-data-based simulations using the probabilities of the transition function. For each policy, 1,000 adolescents were simulated over 28 time points (i.e., 28 challenges were suggested in the simulation). The initial state for the ESM state feature was based on the original distribution of the data on the first day of the study. The simulations were based on the full state space. This means the simulations for the policies using the emotion data are different from the ones with the tiredness data (i.e., due to different transition and reward functions). Therefore, we compare the improvements relative to the random policies in both simulations, rather than directly comparing the performance of using the affect balance feature in the policy compared to using the tiredness feature. To evaluate the adherence when following each policy, the mean adherence across these 1,000 simulations was computed.

To also evaluate diversity within the completed challenges, we set a goal of completing at least 4 challenges of each coping strategy type over the period of 28 days (if the period is longer, a higher number of challenges per coping strategy can be chosen), inspired by Albers et al. (120). For each time point, we measured the progress (a fraction) of reaching this goal. So, completing more than 4 challenges for a specific coping strategy type did not affect the progress, but completing a second challenge of a coping strategy type increased the progress by a fraction of 0.0625. This way, we were able to measure how long it takes to complete 4 challenges of each category for each policy.

As we are interested in the utility of each data feature, we assessed different policies that follow from RL models, including different state features. One policy results from an RL model that uses solely the *C* feature (the deterministic challenge count feature). This feature is only taken into account for the diversity aspect in the reward function ($r_{2}$); therefore, this policy essentially only promotes diversity among the completed challenges. Furthermore, we test policies based on RL models that solely use the *PreDay* feature or the *ESM* feature, and a combination of all state features (which we refer to *full model*). This full model is tested in a myopic and non-myopic setting. Furthermore, we also investigated the added value of adding the cost ($r_{2}$) to promote diversity of completed challenges in the reward function compared to solely promoting adherence ($r_{1}$). The latter analysis was not included in this paper but can be found in the supplementary material and in the analysis code.

#### 4.5.3 Results of the data utility evaluation

**Figure 2:**
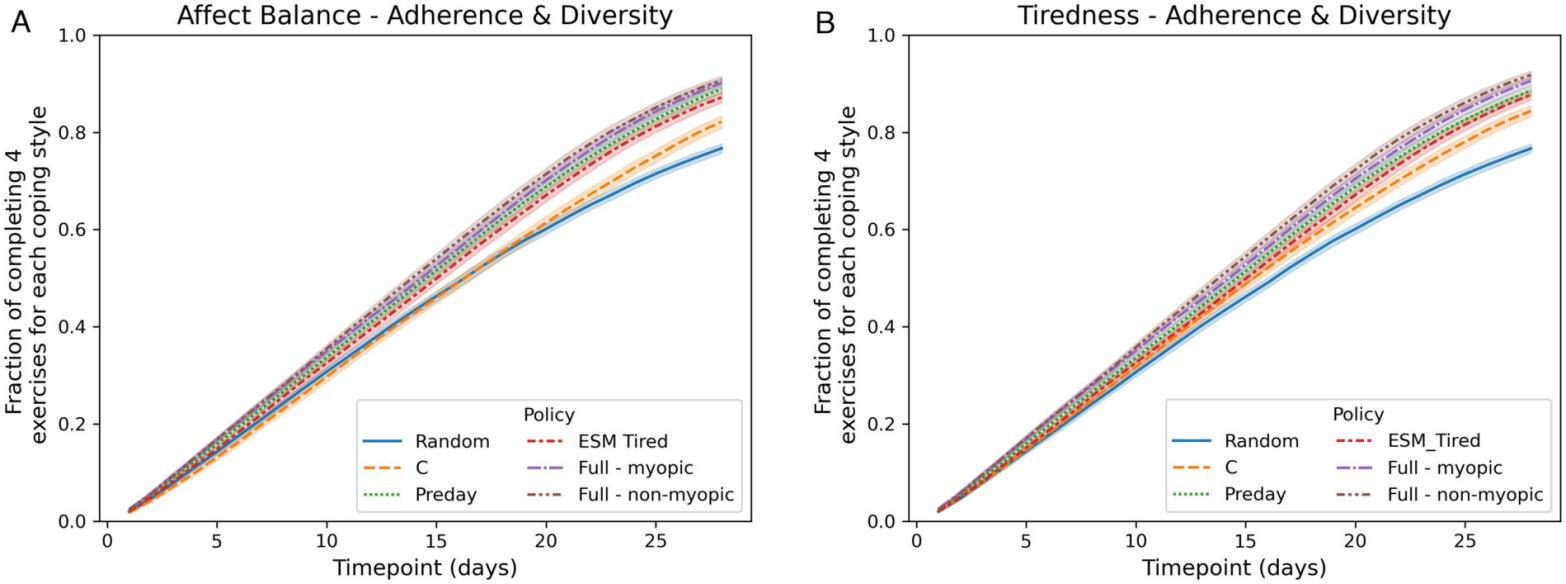
The assessment for diversity within the completed challenges. The mean fractions (with a 95% confidence interval) of completing at least 4 challenges for each coping style over 28 timepoints for **(A)** the affect balance data, and **(B)** the tiredness data. The policy describes the features on which the policy is based. The full policy results from the RL models using all features.

When presenting the results of the simulations, we will first look at the effect of personalization on adherence (i.e., the fraction of completed challenges; Table 1), when promoting both adherence and diversity in the reward function ($r_{1}*r_{2}$). Subsequently, we present the results of our assessment of the diversity of coping strategies within the completed challenges (i.e., completing at least 4 challenges of each coping strategy; Figure 2).

**Table 1:** Simulation results.

Dataset	Affect balance			Tiredness		
Policy	Mean	CI	Improvement	Mean	CI	Improvement
Random	0.528	(0.519, 0.537)	–	0.527	(0.518, 0.536)	–
C	0.501	(0.491, 0.51)	−5.1%	0.518	(0.508, 0.527)	−1.7%
PreDay	0.557	(0.548, 0.565)	5.5%	0.555	(0.546, 0.564)	5.3%
ESM	0.548	(0.539, 0.557)	3.8%	0.547	(0.538, 0.556)	3.8%
Full - myopic	0.568	(0.560, 0.577)	7.6%	0.571	(0.562, 0.579)	8.3%
Full - non-myopic	0.579	(0.57, 0.588)	9.7%	0.587	(0.579, 0.595)	11.4%

The mean fractions of completed challenges (adherence), their 95% confidence intervals (CI), and the improvement in adherence compared to the random policy. The results are based on 1,000 simulated adolescents over a time period of 28 timepoints. The adherence measures for using the different datasets (using affect balance or tiredness) are presented for each policy. The policy describes the features on which the policy is based. The full policy results from the RL models using all features.

##### 4.5.3.1 Assessing adherence

The random policy resulted in a mean adherence fraction of 0.528 (i.e., 52.8% of the suggested challenges were completed) for the affect balance data. The adherence fraction under the random policy could be improved by 5.5% ((0.557−0.528)/0.528∗100) when personalizing on the preday feature, by 3.8% using the affect balance ESM feature, by 7.6% using the full myopic model, and by 9.7% using the full non-myopic model. When using the deterministic count feature (*C*) solely, the adherence drops with 5.1% compared to the random policy. For the tiredness data, the random policy resulted in a mean adherence fraction of 0.527. The adherence could be improved by 5.3% using the preday feature, by 3.8% using the tiredness ESM feature, by 8.3% using the full myopic model, and by 11.4% using the full non-myopic model. When using the deterministic count feature (*C*) solely, the adherence drops with 1.7% compared to the random policy.

##### 4.5.3.2 Assessing diversity

The policies resulting from the RL models all outperform the random policy on the diversity assessment. For the affect balance data, the policy using the count (*C*) feature showed 7% more progress on the diversity assessment at the last timepoint compared to the random policy (Figure 2A). This improvement further increased with 14% using the ESM feature, 16% using the preday feature, 17.5% using the full myopic model, and 18% for the non-myopic model (Figure 2A). The tiredness data showed similar trends, with an improvement of 10% using the count (*C*) feature, 14% using the ESM feature, 15% using the preday feature, 18.1% with the full myopic model, and 20% for the non-myopic model (Figure 2B).

Key Results

• The personalization objective can already be improved by solely using the *Preday* feature and can further be improved by adding the *ESM* feature (the full model containing all features).
• The full non-myopic models perform slightly better than the full myopic models.
• The full models using tiredness data as *ESM* feature perform similarly to the full models using affect balance.

### 4.6 Determine the utility-risk trade-off

In the sixth and final step of our approach, we use the evaluation of the utility of the data features to make an informed decision on what features will be used for the personalization objective. To make this decision, we base the utility on the results that promote the number of completed challenges and the diversity of the different coping strategies within the completed challenges. An overview of the identified risks and the utility can be found in Table 2.

**Table 2:** The utility-risk trade-off.

Data feature	Risks (-) & Risk mitigation (m)	Utility (+)
Preday	∙ Ownership of user data (–, m)	Useful for the personalization objective (+)
Affect balance	∙ Feels personal, private, and sensitive (– –)	Useful for the personalization objective (++)
	∙ Can be confronting (–)	
	∙ Responsibility to act on extreme negative emotions (–, m)	
	∙ Ownership of user data (–, m)	
Tiredness	∙ May feel personal, but less than emotions (–)	Useful for the personalization objective (++)
	∙ Responsibility to act on extreme tiredness (–, m)	
	∙ Ownership of user data (–, m)	

Overview of the identified risks (–), whether they can be mitigated (m), and the utility of the data feature (+). If the risk or utility is considered to be stronger, two symbols are used (++ or – –).

Based on the overview of the identified risks and utility, the least risky option for the personalization objective would be solely using the *Preday* feature. By personalizing using this user feature, we can improve adherence and the diversity of coping strategies compared to a situation with no personalization (Table 1, Figures 2A,B). Furthermore, this option limits the amount of user data that is necessary to operationalize the personalization, and potential associated risks can be mitigated by a mitigation strategy. For instance, by providing users with the possibility to not store their user data in the first place (i.e., opting for a version without personalization using granular consent) and/or delete their already stored data.

To further improve the quality of the personalization objective, tiredness data can be used in combination with the *Preday* feature (Table 1 and Figure 2b). This improvement was similarly to using the emotion data [based on Table 1, the improvement in adherence differs by 1.7% and the diversity assessment differs 2% (20% improvement compared to the random policy; Figures 2a,b)]. Additionally, the ethical risks of tiredness data are considered to be lower than those of emotion data, implying that tiredness data is a suitable alternative. However, using tiredness data does result in more ethical risks than solely using the *Preday* feature, which requires consideration of those risks from the duty of care. They can be handled by deploying mitigation strategies, such as offering appropriate and helpful resources when the adolescent is extremely tired all the time. Additionally, similar mitigation strategies can be used as previously mentioned for the *Preday* feature, providing users with granular consent and agency over their user data. In conclusion, using tiredness data instead of emotion data for the current personalization objective can be deemed an appropriate choice.

## 5 Discussion

Using a case study, we demonstrated a practical precedent for responsible data selection using our ReDS method in the context of personalization algorithms for health apps. The method allows developers of personalization algorithms to (1) make an informed and responsible decision if specific data should be used and (2) improve the transparency of this process. Notably, the ethical implications are dynamic and continue to evolve. Thus, continuous and iterative evaluations of ethical implications are necessary (34). Therefore, the ReDS method requires monitoring over time regarding the made utility-risk trade-off with respect to the personalization object, including revisions on the choices made for the implementation when needed. Furthermore, ethical risks are not quantifiable, making the trade-off between the benefits of specific data features and the ethical risks inherently more subjective and complex. So, instead of paving a means to identify the optimal decision shrouded in subjectivity, our method stimulates reflection on what is considered morally right and promotes decisions in that direction.

The intention of the ReDS method is to be flexible in its application. Although the ReDS method has been demonstrated with a single use case, we believe the method can be applied to other use cases. For instance, by not quantifying ethical reflections, there is flexibility in what ethical considerations may be taken into account, as the relevance of specific ethical considerations is situated in a particular context. The method also provides flexible guidelines for measuring utility, which allows the ReDS method to be applicable to various contexts and different types of algorithms. For instance, there are model-agnostic ways to measure the utility of a specific data feature (e.g., feature importance metrics). Furthermore, the utility–risk trade-off may not always result in a meaningful ethically less sensitive substitute feature, as we found in our presented use case. As described in the stepwise method, other outcomes could be to use the original feature(s) (i.e., when the utility outweighs the risks) or to not deploy any personalization algorithm at all.

To chart a roadmap for the next steps to further refine and evaluate our suggested method, we draw inspiration from the work of Ledo et al. (47), which describes ways to evaluate HCI Toolkit research. In our work, we used a use case *demonstration*. As a next step, other use cases with different contexts should be explored. Although we believe the method itself is generalizable in its applicability, other use cases can inform potential extensions that can allow us to adapt the method to unforeseen situations that may not be currently accounted for. Next, the *usage* can be explored to reveal insights on how developers of personalization algorithms use the method in practice. This can be done by assessing the user experience of developers who used the method using questionnaires or qualitative interviews (47).

Subsequently, if the research community in algorithmic personalization integrates the ReDS method in the way we develop and report on personalization algorithms, a collective understanding of the various ethical risks that may relate to specific types of user data and how the data is being collected (e.g., passive or active). As a result, a repository could be consolidated with several different types of user data, their associated risks, and potential alternative features. This repository might help identify ethical risks, look for alternative features, and improve our collective awareness of ethical implications. When no alternative features are found due to a lack of data availability or other external factors, it might inspire the initiation of new studies that specifically focus on identifying alternative ethically responsible data features. Furthermore, sometimes it may occur that there is no algorithmic—or even technical—solution for the problem that the personalization objective is trying to solve, or no solution can be found in which the utility outweighs the associated ethical risks. In such cases, the conclusion may be that no algorithmic personalization should be applied in the given scenario.

Besides responsible data selection, other elements of the algorithm development process also come with ethical or regulatory risks. For example, the complexity and size of the selected model type can have an impact on the explainability of model outcomes (26, 48, 115), the ease of model development and maintenance (116), and the environmental costs (energy use) (117). While these aspects are not all accounted for in the ReDS method, they are relevant to consider. Future work may focus on translating our method to other aspects of algorithm development and deployment to account for this wider range of ethical implications.

Drawing inspiration from efforts like model cards (39), data nutrition labels (118), data sheets (41), the cognitive biases checklist (42), and the impact assessment card (119), the ReDS method can also be made visible to external stakeholders. It is important for end users and recommenders of mHealth apps (e.g., caregivers, clinicians, and other health professionals) to be able to recognize whether a responsible data-selection approach such as ReDS has been applied. Developers can communicate ReDS adoption using transparent documentation, such as model cards, data sheets, or short ReDS summaries that explicitly report the rationale for data selection, explored alternatives, and ethical trade-offs. Future work can explore the integration of ReDS outcomes into existing app quality and safety assessments, such as conformity assessments under the EU AI Act or other mHealth evaluation frameworks. Developers can employ clear and accessible disclosures within an mHealth app or its accompanying materials, to enable health care professionals and users to understand what data is collected, why it is necessary, and what risks were considered. In this way, ReDS can be made visible and verifiable, thus supporting informed decision-making by both end users and professionals recommending mHealth tools.

## 6 Conclusion

We presented a method for responsible data selection (ReDS) for personalization algorithms for health apps using a case study. Our work offers a practical precedent for developers of personalization algorithms for health apps to explicitly incorporate ethical and regulatory implications into the algorithm development process. The ReDS method promotes responsible engineering practices and allows other developers to learn from the reflections. We hope our work inspires other scholars to conduct more work that implements these ethical and regulatory considerations in data-driven and technical studies and to proactively search for ethically less risky alternative data features. Altogether, our work serves as one of the first steps towards filling the gap from ambition to action.

## Data Availability

The data analyzed in this study is subject to the following licenses/restrictions: Access to the dataset analyzed for the case study presented in this work can be requested via the Grow It! team using a data request form that can be found in the Grow It! codebook. Requests to access these datasets should be directed to the Grow It! codebook: https://osf.io/q83hg?view_only=b691104ecc3d45ad8b48e1bd60ad7125. The code that is used for the presented case study can be found in a repository on 4TU.ResearchData: https://doi.org/10.4121/f65bd258-4179-41ef-8b62-15df5edc8a40.

## Footnotes

^1^https://osf.io/yrqce/?view_only=119840049ad84ded8965ed5e852989a3.

^2^https://doi.org/10.4121/f65bd258-4179-41ef-8b62-15df5edc8a40.
