# Supplementary material for: Responsible data selection method for algorithmic personalization of health apps: a case study on promoting mental health
Source: Front Digit Health. 2026 Jun 24;8:1691697. doi: 10.3389/fdgth.2026.1691697 (PMC13343351; doi:10.3389/fdgth.2026.1691697)
Supplement: Supplementary file 1 [file Datasheet1.pdf]

Supplementary Material

1 SUPPLEMENTARY RESULTS

Here, we present additional results of our analysis, which include simulation results of the reinforcement learning (RL) models that only promote adherence in the reward function. For comparison purposes, Table S1 and Figure 1 also show the results when promoting both adherence and diversity (which are similar to those reported in the main analysis). Our additional analysis demonstrates the added benefit of promoting diversity alongside adherence in the reward function. Similar to the main results of our paper, we first present the results of our assessment of the adherence to the challenges, followed by the diversity assessment.

1.1 Assessing adherence

The random policy resulted in a mean adherence fraction of 0.528 (i.e., 52.8% of the suggested challenges were completed) for the affect balance data (Table S1). When promoting adherence in the reward function, the adherence fraction under the random policy could be improved by 12.3% $((0.593 - 0.528)/0.528 * 100)$ when personalizing on the preday feature, by 11.2% using the affect balance ESM feature, by 15.7% using the full myopic model (preday and affect balance as ESM feature), and by 16.1% when using the full non-myopic model. Based on the results in Table S1, when we also promote diversity in the reward function, we see that promoting diversity comes with a cost for adherence.

For the tiredness data, the random policy resulted in a mean adherence fraction of 0.527. When promoting adherence in the reward function, this fraction could be improved by 12.1% using the preday feature, by 5.9% using the tiredness ESM feature, and by 15.6% using both features in the full myopic model and 16.1% in the full non-myopic model. Similarly to the results with the affect balance data, we see a cost for adherence when also promoting diversity (Table S1).

Table S1. Simulation results. The mean fractions of completed challenges (adherence) and their 95% confidence intervals (CI) as a result of 1000 simulated adolescents over a time period of 28 timepoints. The adherence measures for using the different datasets (using affect balance or tiredness) and different reward functions (adherence, or adherence + diversity) are presented for each policy. The policy describes the features on which the policy is based. The full policy contains the deterministic count feature (C), *Preday* feature, and the *ESM* feature.

**Dataset**	**Affect Balance**				**Tiredness**			
**Reward**	Adherence		Adherence + diversity		Adherence		Adherence + diversity	
**Policy**	Mean	CI	Mean	CI	Mean	CI	Mean	CI
Random	0.528	(0.519, 0.537)	0.528	(0.519, 0.537)	0.527	(0.518, 0.536)	0.527	(0.518, 0.536)
C	-	-	0.501	(0.491, 0.51)	-	-	0.518	(0.508, 0.527)
PreDay	0.593	(0.585, 0.602)	0.557	(0.548, 0.565)	0.591	(0.583, 0.6)	0.555	(0.546, 0.564)
ESM	0.587	(0.577, 0.596)	0.548	(0.539, 0.557)	0.558	(0.549, 0.567)	0.547	(0.538, 0.556)
Full - myopic	0.611	(0.602, 0.619)	0.568	(0.560, 0.577)	0.609	(0.601, 0.618)	0.571	(0.562, 0.579)
Full - non-myopic	0.613	(0.605, 0.622)	0.579	(0.57, 0.588)	0.612	(0.603, 0.62)	0.587	(0.579, 0.595)

1.2 Assessing diversity

When promoting only adherence in the reward function, the random policy outperforms the policies resulting from the other models on the diversity assessment. This is the case for both the affect balance data (with at least 42.1% more progress after 28 timepoints; Figure 1a) and the tiredness data (with at least 14% more progress after 28 timepoints; Figure 1b). So, when solely promoting adherence in the reward function, the coping strategies are not well diversified among the completed challenges. This implies that for our

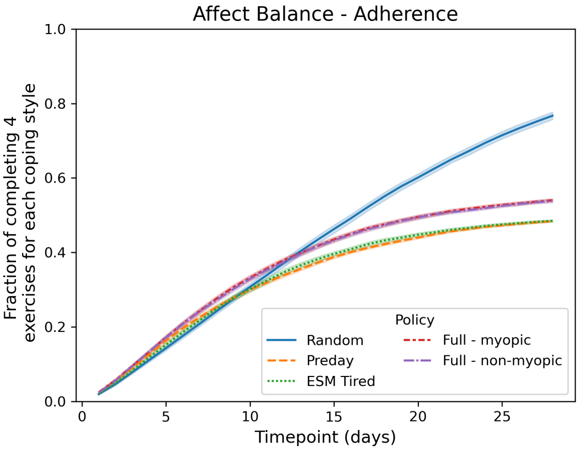

Figure 1a. Promoting adherence (affect balance)

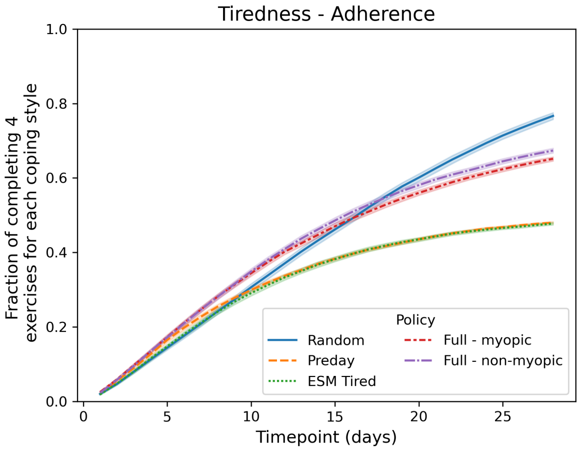

Figure 1b. Promoting adherence (tiredness)

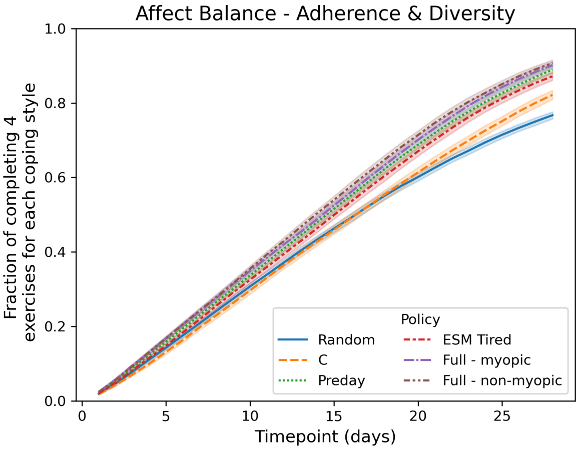

Figure 1c. Promoting adherence + diversity (affect balance)

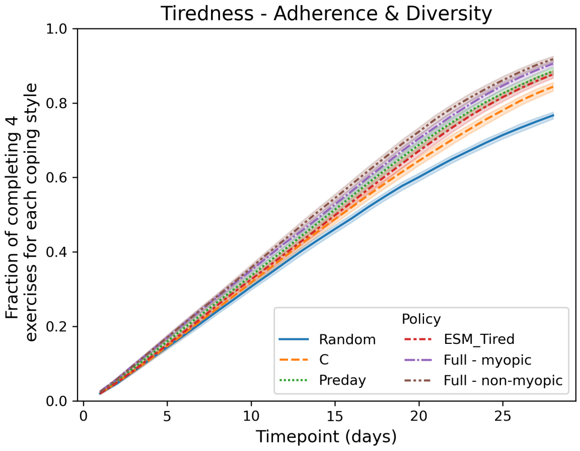

Figure 1d. Promoting adherence + diversity (tiredness)

Figure 1. The assessment for diversity within the completed challenges. The mean fractions (with a 95% confidence interval) of completing at least 4 challenges for each coping style over 28 timepoints for (**a**) the affect balance data and promoting adherence in the reward, (**b**) the tiredness data and promoting adherence in the reward, (**c**) the affect balance data and promoting adherence and diversity in the reward, and (**d**) the tiredness data and promoting adherence and diversity in the reward. The policy describes on which features the policy is based. The full policy contains the deterministic count feature (C in the Figures), *Preday* feature, and the *ESM* feature.

personalization objective (promoting adherence while diversifying coping strategies), solely promoting adherence in the reward function is not sufficient. Indeed, when adding the diversity cost to the reward function, all models outperform the random policy (Figure 1c & Figure 1d).
